# Prognostic Accuracy of Nutritional Assessment Tools in Critically-Ill COVID-19 Patients

**DOI:** 10.3390/jcm14103382

**Published:** 2025-05-13

**Authors:** Mehmet Yildirim, Burcin Halacli, Esat Kivanc Kaya, Ege Ulusoydan, Ebru Ortac Ersoy, Arzu Topeli

**Affiliations:** 1 Department of Internal Medicine, Division of Intensive Care, Faculty of Medicine, Hacettepe University, 06080 Ankara, Türkiye; burcin.halacli@yahoo.com (B.H.); kivanckaya@hacettepe.edu.tr (E.K.K.);; 2 Department of Internal Medicine, Faculty of Medicine, Hacettepe University, 06080 Ankara, Türkiye

**Keywords:** malnutrition, mortality, SARS-CoV-2, mNUTRIC, NRS 2002, MUST

## Abstract

**Objectives**: Critically ill COVID-19 patients are at high risk of malnutrition; however, no study has directly compared the prognostic accuracy of different nutritional assessment tools. This study aimed to determine the optimal cutoff values for the Modified Nutrition Risk in the Critically Ill (mNUTRIC) score, Nutritional Risk Screening 2002 (NRS 2002), and Malnutrition Universal Screening Tool (MUST) and to evaluate their predictive value for ICU mortality. **Method**: A retrospective analysis was conducted on patients with laboratory-confirmed COVID-19 admitted to our ICU between 20 March 2020 and 15 June 2021. Clinical and laboratory data, as well as patient outcomes, were retrieved from electronic medical records and patient charts. The mNUTRIC, NRS 2002, and MUST scores were calculated at ICU admission. **Results**: The study included 397 patients, with 273 survivors and 124 non-survivors. The median age was 65 (55–76) years, and the median BMI was 26.1 (24.0–29.4). Non-survivors had significantly higher median scores in all three nutritional assessment tools compared to survivors (mNUTRIC: 5 vs. 3, NRS 2002: 4 vs. 3, MUST: 2 vs. 2; *p* < 0.01). At the optimal cutoff values, mNUTRIC ≥ 4 demonstrated the highest prognostic accuracy (sensitivity: 0.77, specificity: 0.74; AUC = 0.75, CI = 0.70–0.81), followed by NRS 2002 ≥ 4 (sensitivity: 0.63, specificity: 0.60; AUC = 0.62, CI = 0.56–0.67) and MUST ≥ 3 (sensitivity: 0.21, specificity: 0.91; AUC = 0.56, CI = 0.50–0.68). Higher scores were associated with increased disease severity, poorer patient performance, prolonged hospital stays, and elevated ICU, 28-day, and overall hospital mortality rates. Among the three assessment tools, only an mNUTRIC score of ≥ 4 was independently associated with ICU mortality (OR = 1.54, CI = 1.21–1.96, *p* < 0.01). **Conclusions**: At ICU admission, mNUTRIC ≥ 4, NRS 2002 ≥ 4, and MUST ≥ 3 were identified as the most accurate predictors of mortality in critically ill COVID-19 patients. However, only the mNUTRIC score was an independent predictor of ICU mortality.

## 1. Introduction

In critically ill patients, poor nutritional status is independently associated with longer length of stay (LOS), longer duration of mechanical ventilation (MV), higher mortality rates, secondary infections, long-term poor outcomes and higher cost [1,2]. Since critically ill patients are generally in a proinflammatory catabolic state during the early acute illness phase, the results of impaired nutritional status are likely to be more exaggerated. Also, acute illness itself may impair nutritional status and contribute to poor outcomes [3].

Coronavirus disease 2019 (COVID-19) is characterized by variable clinical features ranging from asymptomatic illness to acute respiratory distress syndrome (ARDS) [4,5]. Approximately 5% of the COVID-19 patients and 20% of those hospitalized experience severe COVID-19 symptoms and require intensive care [6,7,8]. The severe disease involves ARDS, hyperinflammation, multiorgan failure, acute kidney injury (AKI), cardiovascular dysfunction, coagulopathy or neurologic disorders [9]. Advanced age, comorbidities such as diabetes, hypertension and lung diseases, immunosuppression, malignancy and obesity are some of the poor prognostic factors in patients with COVID-19 [10,11,12,13]. Since the 1918 influenza pandemic, malnutrition has become a common condition and a well-known risk factor for poor prognosis and mortality in patients with severe viral pneumonia and acute respiratory failure [14]. Some recent studies have confirmed these findings in critically ill COVID-19 patients [15,16,17]. During the viral infection course, increased synthesis of proinflammatory markers such as C-reactive protein (CRP), tumor necrosis factor-alpha (TNF-α), ferritin and interleukins increase consumption of albumin and muscle protein, which is the main cause of susceptibility to malnutrition [18]. Furthermore, in most of the COVID-19 patients, gastrointestinal symptoms (diarrhea, nausea, vomiting, anorexia) accompany respiratory symptoms, which may contribute to impaired nutritional status [19,20].

Several screening tools have been developed and are widely used in clinical practice to identify patients at nutritional risk. Among them, the Modified Nutrition Risk in the Critically Ill (mNUTRIC) score [21], Nutritional Risk Screening 2002 (NRS 2002) [22] and Malnutrition Universal Screening Tool (MUST) [23] are the most common, and they have been developed to predict malnutrition for outpatients and inpatients. The mNUTRIC score, initially proposed by Heyland et al. [24], was developed specifically for ICU patients. It incorporates objective illness severity measures—such as age, Acute Physiology and Chronic Health Evaluation (APACHE) II score, Sequential Organ Failure Assessment (SOFA) score, number of comorbidities, and days from hospital to ICU admission—and aims to identify patients most likely to benefit from nutritional interventions. Unlike traditional tools, mNUTRIC omits anthropometric or dietary intake variables, making it feasible in sedated or ventilated patients. However, it may fail to detect pre-existing chronic malnutrition, as it does not include parameters like recent weight loss or reduced oral intake [21,25].

To address nutritional risk in general hospitalized populations, NRS 2002 was developed by the ESPEN working group and includes components such as BMI, recent weight loss, decreased food intake, and disease severity [22]. It is one of the most validated tools in both surgical and medical inpatients [26]. While it captures recent nutritional deterioration, its application in ICU settings is limited by the subjective nature of dietary history, which is often unavailable or unreliable in sedated patients [27].

The MUST tool, recommended for use in community and hospital settings, is based on BMI, unintentional weight loss, and the effect of acute disease [23]. Its simplicity and wide applicability make it popular, especially in screening large populations [28]. However, similar to NRS 2002, MUST relies heavily on patient cooperation and historical documentation. In ICU patients—especially those intubated or unconscious—accurate assessment of recent intake or weight loss is often challenging, leading to potential under- or overestimation of risk [29].

According to the literature, mNUTRIC, NRS 2002 and MUST score higher than 5, 2 and 1, respectively, are considered as high scores and reflect nutritional risk. However, comparative studies have shown moderate agreement between these tools in ICU settings, but they may identify different subsets of patients at risk due to varying constructs [1]. For instance, a multicenter study by Mukhopadhyay et al. [25] reported that mNUTRIC, but not NRS 2002 or MUST, was independently associated with 28-day mortality. Another study by de Vries et al. [30] confirmed the superiority of mNUTRIC in predicting adverse outcomes in mechanically ventilated ICU patients.

Despite their widespread use, none of these tools have been specifically validated for COVID-19 populations, which further underscores the need for comparative studies evaluating their predictive accuracy in critically ill COVID-19 patients. Therefore, the European Society for Clinical Nutrition and Metabolism (ESPEN) guideline on clinical nutrition in the intensive care unit [31] recommends general clinical assessment to assess nutritional status until a specific tool has been validated. At the beginning of the COVID-19 crisis in 2020, ESPEN also released expert statements and practical guidance for nutritional management [32], which suggests checking NRS 2002 and MUST for hospitalized COVID-19 patients and NUTRIC score for patients in intensive care unit (ICU). Since then, there have not been any validated nutritional assessment tools to screen the nutritional status of those patients

In this study, we aimed to determine optimum thresholds of mNUTRIC score, NRS 2002 and MUST on ICU admission for predicting ICU mortality in critically ill COVID-19 patients. We also aimed to evaluate and compare the prognostic accuracy of these nutritional assessment tools.

## 2. Methods

The study was approved by the Hacettepe University Non-interventional Clinical Research Ethics Board (reference number: GO 22/277 Date:15 March 2022), and the approval of the Turkish Ministry of Health was obtained.

### 2.1. Patients Selection

A retrospective observational study was conducted on critically ill adult patients (aged > 18 years) with laboratory-confirmed COVID-19, defined as a positive SARS-CoV-2 polymerase chain reaction (PCR) test from a nasopharyngeal-oropharyngeal swabs or tracheal aspirate samples. All patients included in the study required intensive care unit (ICU) admission. Patients under 18 years of age and those without laboratory confirmation of COVID-19 were excluded from the study. We reviewed the records of COVID-19 patients who had been admitted to our medical ICU between 20 March 2020 and 15 June 2021.

### 2.2. Data Collection

Data were collected from electronic medical records and patient charts. Demographic data, comorbidities, patient performance scores (Eastern Cooperative Oncology Group (ECOG) Performance Status, Clinical Frailty Scale (CFS)), disease severity scores (APACHE II, SOFA) on admission and referral center (emergency room, ward, other center) were recorded. Laboratory values, arterial blood gas analysis, partial pressure of arterial oxygen/fraction of inspired oxygen ratios (PaO_2_/FiO_2_) on ICU admission, and invasive mechanical ventilation (IMV) requirement were noted. The presence of septic shock was based on Sepsis-3 definitions [33], and acute kidney injury (AKI) was defined by Kidney Disease Improving Global Outcomes criteria [34]. To determine BMI, the body weight (kilogram = kg) and height (meter = m) of the patients, which were measured by nurses on ICU admission, were noted, and BMI was calculated using the formula: weight (kg)/height^2^ (m^2^). Based on the World Health Organisation (WHO) BMI classification [35], patients were categorized into four groups: underweight: BMI < 18.50, normal weight: BMI = 18.50–24.99, overweight: BMI = 25–29.99, obese: BMI ≥ 30.d

#### 2.2.1. Nutritional Risk Assessment Tools and Outcomes

##### mNUTRIC, NRS 2002, MUST

Age, APACHE II score, SOFA score, number of comorbidities and days from hospital admission to ICU admission were recorded to calculate the mNUTRIC score [21]. Information on unplanned weight loss in the last 1, 2 and 3 years and reduced oral intake of patients were searched from patients’ electronic records, which was noted by doctors on ICU admission. NRS 2002 scores were calculated as previously defined by Kondrup et al. [22]. In addition to these data, to determine the MUST score [23], we also scanned patients’ charts to access information on whether there has been no nutrition intake for more than 5 days.

#### 2.2.2. Outcomes

ICU mortality was the outcome analyzed in this study, while 28th-day mortality, hospital mortality, ICU length of stay (LOS), and hospital LOS were also recorded.

### 2.3. Statistical Analysis

Descriptive statistics were presented using median and interquartile range (25th–75th percentiles—IQR) for continuous variables; frequency and percent for categorical variables and comparisons between independent subgroups were performed using the Mann–Whitney U test and Chi-square test, respectively. The predictive values of prognostic factors for ICU mortality were analyzed using receiver-operator characteristics (ROC) analyses. Cutoff values were determined based on the Youden index [36] to maximize sensitivity and specificity. For multivariate analysis, clinically relevant significant variables detected from univariate analysis were further entered into the binary logistic regression analysis to determine independent predictors of ICU mortality. A type-I error level of 5% was considered a statistical significance threshold. All analyses were performed with the SPSS 23 IBM^®^ statistics program (IBM Inc., Armonk, NY, USA).

## 3. Results

### 3.1. Characteristics of Patients

A total of 397 patients were analyzed. Baseline characteristics, outcomes and related laboratory values on the admission of ICU survivors (*n* = 273) and non-survivors (*n* = 124) were shown in the Appendix A (Table A1). The median age of all patients was 65 (55–76) years, and ICU survivors were younger than non-survivors (*p* < 0.01). The frequency of hypertension (*p* = 0.02), cardiac disease (*p* < 0.01), and malignancy (*p* < 0.01) were higher in non-survivors than in other patients. Among the 77 patients diagnosed with malignancy, the distribution of malignancy types was as follows: hematologic malignancies in 19 patients and solid tumors in 58 patients. The solid tumors included gastrointestinal (*n* = 15), lung (*n* = 14), urinary system (*n* = 9), gynecological *(n* = 7), breast (*n* = 5), central nervous system (*n* = 3), thyroid (*n* = 3), and skin cancers (*n* = 2). Additionally, 18 of 77 patients were identified as having advanced-stage cancer. The median BMI of all patients was 26.1 (24.0–29.4), and there was no difference between survivors and non-survivors (26.5 vs. 25.7; *p* = 0.09) while 97 (35.5%) of the survivors and 67 (46.0%) of the non-survivors had a BMI lower than 25 (*p* = 0.03). Regarding nutritional assessment tools, median mNUTRIC, NRS 2002 and MUST scores were higher in non-survivors than other patients (5 vs. 3, 4 vs. 3 and 2 vs. 2, respectively; *p* < 0.01). ICU, 28 days and hospital mortality rates were 31.2%, 24.2% and 33.5%, respectively.

### 3.2. Analysis of Nutrition Assessment Tools

Based on the current literature, patients who are at malnutrition risk on ICU admission were analyzed. As the current literature suggests, a mNUTRIC score equal to or greater than 5, an NRS 2002 score equal to or greater than 4, and a MUST score equal to or greater than 2 reflect malnutrition risk these thresholds were used (Table 1). Among the 397 critically ill COVID-19 patients included in the study, 103 patients (25.9%) were identified as being at high nutritional risk according to the mNUTRIC score (≥5), while 187 patients (47.1%) were considered high risk based on the NRS 2002 score (≥4). Because the presence of acute illness leads to a 2-point increase in MUST score (acute disease effect score), all of our patients have at least 2 points.

Patients in the high-risk group defined by both tools were significantly older (median age: 77 years in both groups) and had a greater burden of comorbidities. For instance, the prevalence of hypertension was 72.8% in the mNUTRIC high-risk group and 63.1% in the NRS 2002 group. Similarly, malignancy was more common among high-risk patients (32.0% in mNUTRIC and 25.7% in NRS 2002). The median ECOG score was 2 in both, and the CFS score was 6 in the mNUTRIC high-risk group and 5 in the NRS 2002 high-risk group. APACHE II scores were 22 [18–27] in the mNUTRIC high-risk group and 17 [14–21] in the NRS 2002 high-risk group, while median SOFA scores were 4 in both. The frequency of IMV need on ICU admission was 74.8% (mNUTRIC high-risk group) and 48.1% (NRS 2002 high-risk group). ICU mortality was 63.1% in the mNUTRIC high-risk group and 41.7% in the NRS 2002 group. Hospital mortality reached 66.0% and 44.9%, and 28-day mortality was 49.5% and 33.2%, respectively. The median length of stay in the ICU was longer in the mNUTRIC high-risk group (14 vs. 11 days total), and the hospital length of stay was 23 days in the mNUTRIC group and 22 days in the NRS 2002 group.

**Figure 1:**
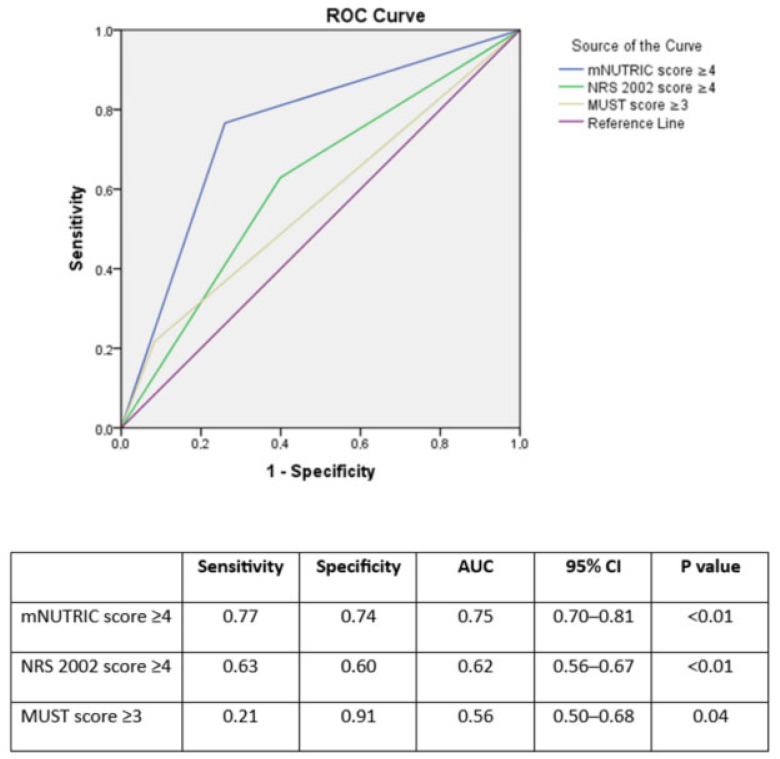
Receiver operating characteristic curve (ROC) for prediction of intensive care unit mortality based on the nutrition assessment tools. mNUTRIC: Modified Nutrition Risk in the Critically Ill score, NRS 2002: Nutritional Risk Screening 2002, MUST: Malnutrition Universal Screening Tool.

Based on Youden index [36], at a threshold of 4 or greater mNUTRIC (sensitivity: 0.77 and specificity: 0.74; AUC = 0.75 CI = 0.70–0.81), NRS 2002 scores (sensitivity: 0.63 and specificity: 0.60; AUC = 0.62 CI = 0.56–0.67) and at a threshold of 3 or greater MUST score (sensitivity: 0.94 and specificity: 0.21; AUC = 0.56 CI = 0.50–0.68), had the best prognostic accuracy (*p* < 0.01) (Table 2). The receiver operating characteristic curve (ROC) for prediction of intensive care unit mortality based on the nutritional assessment tools is shown in Figure 1.

Patients were divided into two groups according to nutritional assessment tools’ thresholds and features, and the outcomes of these groups were analyzed (Table 3). For all these three nutritional assessment tools, patients who had a higher or equal score than thresholds were older (75 [66–82] vs. 59 [51–67] for mNUTRIC (*p* < 0.01); 77 [71–82] vs. 57 [51–63] for NRS 2002 (*p* < 0.01); 66 [53–74] vs. 65 [55–76] for MUST (*p* = 0.77)) and had worse disease severity (APACHE II and SOFA) and patient performance scores (ECOG and CFS) (*p* < 0.01). There was no difference in median BMI values in patients with a mNUTRIC score < 4 (26.0 [23.4–29.4]) and ≥4 (26.1 [24.2–29.4]) (*p* = 0.21) whereas patients with an NRS 2002 score ≥ 4 or MUST score ≥ 3 had lower median BMI values than other patients (25.5 [23.1–29.3] vs. 26.9 [24.6–29.5] and 21.2 [19.0–25.2] vs. 26.5 [24.3–29.5], respectively (*p* < 0.01 for both). According to the mNUTRIC score, patients with scores ≥ 4 had a significantly higher ICU mortality rate compared to those with mNUTRIC < 4 (57.2% vs. 12.6%, *p* < 0.01), as well as significantly increased 28-day mortality (45.2% vs. 9.1%, *p* < 0.01) and hospital mortality (59.6% vs. 14.7%, *p* < 0.01). Similarly, patients at nutritional risk based on the NRS 2002 score (≥4) had worse outcomes than those with lower scores. ICU mortality was 41.7% vs. 21.9% (*p* < 0.01), 28-day mortality was 33.2% vs. 16.2% (*p* < 0.01), and hospital mortality was 44.9% vs. 23.3% (*p* < 0.01). When patients were stratified according to the MUST score, those with scores ≥ 3 also had significantly higher rates of death. ICU mortality was 54.0% vs. 28.0% in patients with MUST < 3 (*p* < 0.01), 28-day mortality was 40.0% vs. 21.9% (*p* < 0.01), and hospital mortality was 60.0% vs. 29.7% (*p* < 0.01).

Laboratory values of patients on ICU admission according to nutritional risk scores’ thresholds were depicted in Table 4. Patients with mNUTRIC ≥ 4 had significantly lower median prealbumin levels (11 [8–15] mg/dL) compared to those with mNUTRIC < 4 (13 [9–18] mg/dL, *p* < 0.01). A similar pattern was observed with the NRS 2002 score, where prealbumin levels were 11 [8–15] mg/dL in high-risk patients and 13 [10–18] mg/dL in low-risk patients (*p* < 0.01). However, no statistically significant difference was observed in prealbumin levels between MUST ≥ 3 and <3 groups (*p* = 0.08). Albumin levels were significantly lower in patients with higher nutritional risk across all three tools. For mNUTRIC ≥ 4 vs. <4, the median albumin was 3.0 [2.7–3.4] vs. 3.3 [3.0–3.6] g/dL (*p* < 0.01); for NRS 2002 ≥ 4 vs. <4, 3.1 [2.7–3.5] vs. 3.3 [3.0–3.6] g/dL (*p* < 0.01); and for MUST ≥ 3 vs. <3, 3.2 [2.8–3.5] vs. 3.3 [2.9–3.6] g/dL (*p* = 0.01). Total protein levels were also lower in high-risk groups according to mNUTRIC (6.1 [5.5–6.6] vs. 6.3 [5.8–6.8] g/dL, *p* < 0.01) and MUST (6.2 [5.7–6.7] vs. 6.3 [5.8–6.8] g/dL, *p* = 0.04), but not significantly different between NRS 2002 risk groups (*p* = 0.30). Blood urea nitrogen (BUN) levels were significantly higher in patients at nutritional risk according to both mNUTRIC and NRS 2002 scores (mNUTRIC ≥ 4: 28.7 [19.2–48.3] vs. <4: 17.7 [13.8–23.1] mg/dL, *p* < 0.01; NRS 2002 ≥ 4: 24.6 [18.0–40.4] vs. <4: 17.5 [13.5–24.7] mg/dL, *p* < 0.01). No significant difference was found in BUN levels between MUST groups (*p* = 0.50). Procalcitonin levels were significantly higher among patients with high mNUTRIC scores (0.47 [0.14–1.91] vs. 0.21 [0.10–0.45] ng/mL, *p* < 0.01) and NRS 2002 ≥ 4 (0.32 [0.11–1.22] vs. 0.25 [0.10–0.67] ng/mL, *p* < 0.01). A statistically significant difference was also observed between MUST ≥ 3 and <3 groups (0.25 [0.10–0.80] vs. 0.22 [0.09–0.72] ng/mL, *p* = 0.02).

## 4. Independent Variables for Predicting ICU Mortality

In the multivariate logistic regression analyses conducted to identify independent predictors of ICU mortality, three separate models were evaluated, each incorporating a different nutritional risk screening tool (mNUTRIC, NRS 2002, or MUST) along with common clinical covariates. In Model A, which included the mNUTRIC score (≥4), this parameter was also found to be a statistically significant independent predictor of ICU mortality (OR: 1.49, 95% CI: 1.23–1.88, *p* = 0.02). Conversely, in Model B (including NRS 2002 ≥ 4) and Model C (including MUST ≥ 3), the nutritional screening variables were not statistically significant (*p* = 0.94 and *p* = 0.22, respectively). Across all models (A, B, and C), malignancy, CFS, and IMV on admission were consistently identified as statistically significant independent predictors of ICU mortality (all *p* < 0.05). All three models demonstrated good model fit based on the Hosmer and Lemeshow test (Model A: *p* = 0.63; Model B: *p* = 0.41; Model C: *p* = 0.57) and explained a similar proportion of variance in ICU mortality (Nagelkerke R^2^ = 0.68 for all models) (Table 5).

## 5. Discussion

Our study revealed that at a threshold equal to or greater than 4 for mNUTRIC and NRS 2002 scores, equal to or greater than 2 for MUST score, have the best prognostic accuracy on ICU mortality. We also showed that patients who had higher or equal scores than these thresholds had worse ICU outcomes. To the best of our knowledge, the present study is the first study that evaluates the prognostic accuracy of these three nutritional assessment tools together.

Malnutrition in the hospital setting is a well-known risk factor for adverse outcomes [37]. Severe SARS-CoV-2 infection predisposes to poor nutritional status, leading to catabolic inflammatory response, reduced food intake due to dysgeusia and anosmia, gastrointestinal system symptoms and physical immobilİty. Poor nutritional status in COVID-19 patients results in micronutrient deficiency, a decrease in lean body mass and an impaired immune response that aggravates disease severity [28,38]. Vong et al. [28] retrospectively analyzed 4311 COVID-19 adult hospitalized patients, 2931 of whom were followed in ICU, and reported a 76% malnutrition prevalence in these patients according to MUST score. In that previous study, malnutrition was independently associated with higher mortality rates and longer hospital LOS in critically ill COVID-19 patients. In our study, a MUST score of 3 is the best threshold to predict ICU mortality. In the literature, we came across only one study by Liu et al. [29], which evaluated the prognostic, predictive accuracy of MUST score in hospitalized COVID-19 patients. Liu et al. [29] investigated 141 elderly patients with COVID-19 (95 severe or very severe COVID-19) and reported a 0.72 sensitivity rate to predict hospital LOS for a MUST score ≥ 2, but ICU or hospital mortality was not analyzed as the outcome. The present study was performed in a larger cohort than the previous study [29] and showed that the MUST score is also associated with ICU mortality in adult critically ill COVID-19 patients. Although the MUST score showed a relatively high sensitivity in predicting ICU mortality at a threshold of ≥3, it demonstrated a low specificity, resulting in a higher false-positive rate. This finding is consistent with previous reports indicating that the MUST score, originally developed for community and general hospital settings, may not fully capture the complexity of nutritional risk in critically ill patients [23,31]. The tool incorporates components such as BMI, recent weight loss, and acute disease effect, which can be significantly influenced by fluid overload, immobilization, or pre-existing sarcopenia in ICU patients, thereby reducing specificity [26].

In addition, MUST does not account for acute physiological parameters or illness severity—factors that are highly relevant for predicting mortality in ICU settings. This likely explains why, although MUST was significantly associated with ICU mortality in univariate analysis, it did not remain an independent predictor in the multivariable regression model. In contrast, tools like mNUTRIC, which integrate markers of acute illness (e.g., APACHE II and SOFA scores), have demonstrated stronger associations with ICU outcomes and have been validated in critically ill populations [39]. These observations support the notion that while MUST can serve as a practical and sensitive screening tool, especially during the early phases of hospitalization, its prognostic value in ICU settings may be limited due to its design and inherent constraints.

NRS 2002 is one of the most important nutritional assessment tools, and it is based on three components: age, nutritional status, and disease severity [22]. American Society for Parenteral and Enteral Nutrition (ASPEN) [40] suggests that all patients in the ICU should be assessed for nutritional risk using the NRS 2002 or NUTRIC score. In an expert opinion, patients who have an NRS 2002 score equal to or higher than 3 indicate a risk for malnutrition. However, NRS 2002 was not originally developed for ICU patients, and few previous studies have investigated the prognostic predictive accuracy of NRS 2002. In 2018, Macial et al. [27] conducted a prospective cohort study in 185 critically ill patients to examine the association between NRS 2002 thresholds and ICU outcomes. ICU and hospital mortality were higher in patients with high nutritional risk than in patients with nutritional risk, while there were no differences in ICU and hospital LOS between the two groups. Although the previous study [27] showed higher ICU mortality in patients with high risk, there was no analysis for determining the best NRS 2002 threshold to predict ICU outcomes. Alikiaii et al. [17] evaluated 73 critically ill COVID-19 patients and found a strong correlation (OR = 34.5, CI = 5.2–228.3) between NRS 2002 score and mortality. In another previous study on 285 ICU patients, an NRS 2002 score ≥ 3 was independently associated with ICU mortality (OR = 2.3, CI = 1.0–5.0). For the NRS 2002 score, a threshold equal to or greater than 4 had the best prognostic accuracy to predict ICU mortality. Due to the fact that the previous studies in the literature did not examine the prognostic, predictive accuracy of different thresholds of NRS 2002 in critically ill COVID-19 patients, we were not able to compare our findings.

The NUTRIC score, which reflects poor nutritional status and inflammation, consists of six parameters: age, APACHE II score, SOFA score, comorbidities, days from hospitalization to ICU admission, and the interleukin-6 (IL-6) level [24]. However, the use of the NUTRIC score remains limited due to the unavailability of IL-6. mNUTRIC score was developed to overcome this limitation, and Rahman et al. validated the mNUTRIC score as an independent predictor for mortality [21]. Other previous studies have also reported that a mNUTRIC score ≥5 indicates high nutritional risk and is associated with ICU mortality and higher LOS [25,41,42]. On the other hand, there have been some studies that found different cutoff values for the mNUTRIC score. A single-center retrospective study from the Netherlands [30] was performed among 475 mechanical ventilated patients, and according to the Youden index, the best cut-off value was ≥5 to predict 28^th^-day mortality. Similar to this previous study, a secondary analysis of The Beijing Acute Kidney Injury Trial (BAKIT) [42], which evaluated 3107 critically ill patients, found a mNUTRIC score ≥ 5 had the best Youden index (sensitivity 61.5% and specificity of 78.8%) to predict 28th-day mortality. Another previous study from South Korea [43] revealed the best cutoff value as 6 (sensitivity 75% and specificity 65%) for 28th-day mortality. Tseng et al. [44] conducted a multicenter retrospective study in patients with severe pneumonia, and the optimal cutoff value to predict treatment failure for pneumonia was ≥6 (sensitivity of 78.6% and specificity of 76.2%). In our study, according to the Youden index, a threshold of 4 or greater had the best prognostic accuracy in predicting ICU mortality. To the best of our knowledge, our study was the first one that aimed to identify the threshold value for mNUTRIC score in critically ill COVID-19 patients.

The divergence in predictive performance among the three assessment tools evaluated in this study may be attributed to their underlying design features and intended populations. Both NRS 2002 and MUST were originally developed for use in general hospital or outpatient populations, and they primarily rely on anthropometric parameters and recent dietary intake or weight loss. In ICU patients—particularly those who are sedated, ventilated, or experiencing acute fluid shifts—these variables may be difficult to assess accurately or may not reflect current physiological status. This can lead to misclassification and reduced prognostic accuracy, which may explain why these tools did not retain independent predictive value for ICU mortality in our multivariable analysis. Additionally, these tools do not incorporate markers of systemic inflammation or organ dysfunction, which are highly relevant in the pathogenesis of malnutrition in critically ill populations. A recent large-scale multicenter study by Chen et al. demonstrated a significant association between malnutrition and elevated systemic immune-inflammation index (SII) in hospitalized patients, emphasizing the critical role of inflammatory burden in nutritional risk [45]. This further supports the limited applicability of tools that fail to account for acute-phase response in ICU settings. In contrast, the mNUTRIC score includes ICU-specific variables such as APACHE II and SOFA scores, as well as age and comorbidities, all of which are established predictors of adverse outcomes. By integrating acute illness severity, mNUTRIC offers a more dynamic and context-sensitive risk stratification, likely accounting for its superior performance in our cohort. These findings underscore the importance of selecting nutritional assessment tools that are tailored to the clinical setting and the characteristics of the target population.

The clinical implications of sensitivity and specificity are essential when interpreting the utility of nutritional screening tools in the ICU. A high sensitivity, as observed with MUST and NRS 2002 in our study, suggests that these tools are effective in identifying a large proportion of patients at potential nutritional risk. This characteristic makes them suitable for broad screening approaches to avoid missing vulnerable individuals. However, their lower specificity increases the risk of false-positive classifications, potentially resulting in unnecessary allocation of nutritional resources [46]. On the other hand, the mNUTRIC score, which demonstrated a more balanced sensitivity and specificity, is designed specifically for critically ill patients and includes illness severity variables. This makes it particularly effective for prioritizing patients who are most likely to benefit from intensive nutritional intervention, thus improving the precision of nutritional care in the ICU setting [21]. Recent clinical recommendations emphasize the need to tailor the use of screening tools to the goals of care. While tools with high sensitivity are useful for early case finding, those with higher specificity are essential when therapeutic decisions or resource-intensive interventions are considered [26].

Our findings also suggest that age and comorbidities (hypertension, cardiac disease, malignancy) may have influenced the predictive performance of the nutritional screening tools evaluated in this study. The mNUTRIC score includes both age and the number of comorbidities as scoring components, which may partly explain its superior prognostic accuracy in predicting ICU mortality [47]. In contrast, NRS 2002 and MUST do not directly incorporate comorbidity burden or chronological age into their algorithms. Given that older age and the presence of multiple comorbidities (e.g., malignancy, cardiovascular disease, diabetes) were more common in non-survivors, it is plausible that tools accounting for these factors are more reflective of overall physiological vulnerability in critically ill COVID-19 patients [11,48]. This reinforces the relevance of mNUTRIC as a context-appropriate screening tool for ICU populations, particularly in settings where age and comorbidity burden are strong determinants of outcome.

Our findings may help improve how nutritional support is planned in the ICU. Since the mNUTRIC score showed better predictive accuracy than other tools, it could be used to guide more focused and timely nutritional interventions. Using mNUTRIC at ICU admission may help identify patients who need urgent nutritional support while avoiding unnecessary treatment in lower-risk patients [42,49]. In contrast, tools like NRS 2002 and MUST, though useful in general wards, may be less reliable in ICU patients due to their lack of illness severity markers. In practice, a two-step approach could be considered—starting with a sensitive screening tool like NRS 2002 at hospital admission, followed by mNUTRIC in critically ill patients to guide ICU-specific decisions. Including these tools in electronic health records or clinical protocols could also help streamline care and make nutrition assessment more consistent [50].

The results of the present study should be interpreted in the light of several limitations. First, because of its retrospective nature, information on unplanned weight loss and reduced oral intake of patients was obtained from patients’ electronic records, which were routinely noted by doctors on ICU admission. Therefore, this information might have been missing, which led to an underestimation of NRS 2002 and MUST scores. The second limitation of the study is the lack of data on the patients’ actual daily caloric and protein intake during their ICU stay. Although medical nutrition therapy was routinely provided in accordance with international guidelines, detailed nutritional intake records were not available due to the retrospective nature of the study. Third, this was a single-center cohort study that needs to be validated and reproduced by further multicenter studies in larger cohorts, which would increase the generalizability of our findings. Finally, we have analyzed critically ill patients with COVID-19. Therefore, our results may not reflect all critically ill patients.

## 6. Conclusions

On ICU admission, mNUTRIC score ≥ 4, NRS 2002 score ≥ 4 and MUST score ≥ 2 had the best prognostic accuracy in critically ill COVID-19 patients. Among these nutritional assessment tools, only mNUTRIC score was independently associated with ICU mortality. Further studies in larger patient cohorts are needed to confirm our findings.

## Figures and Tables

**Table 1:** Characteristics and Outcomes of Patients at Malnutrition Risk according to mNUTRIC and NRS 2002.

	Total (n = 397)	Patients at Malnutrition Risk According to mNUTRIC (mNUTRIC ≥ 5) (n = 103, 25.9%)	Patients at Malnutrition Risk According to NRS 2002 (NRS 2002 ≥ 4) (n = 187, 47.1%)
Age *, years	65 [55–76]	77 [68–82]	77 [71–82]
**Male sex, n (%)**	254 (64.0)	61 (59.2)	117 (62.6)
**Comorbidities, n (%)**			
Hypertension	209 (52.6)	75 (72.8)	118 (63.1)
Diabetes	136 (34.3)	46 (44.7)	72 (38.5)
Cardiac disease	134 (33.8)	59 (57.3)	85 (45.5)
Malignancy	77 (19.4)	33 (32.0)	48 (25.7)
Chronic lung disease	66 (16.6)	20 (19.4)	35 (18.7)
Chronic kidney disease	35 (8.8)	19 (18.4)	21 (11.2)
ECOG *	1 [0–2]	2 [2–3]	2 [1–3]
CFS *	3 [2–6]	6 [4–7]	5 [3–7]
APACHE II score *	15 [11–19]	22 [18–27]	17 [14–21]
Admission SOFA score *	4 [2–5]	4 [3–6]	4 [3–6]
PaO_2_/FiO_2_ on admission *	150 [113–226]	134 [101–187]	154 [118–230]
**IMV on admission, n (%)**	162 (40.8)	77 (74.8)	90 (48.1)
Duration of IMV, days *	12 [5–22]	12 [5–21]	13 [7–22]
**AKI on admission, n (%)**	112 (28.2)	54 (52.4)	64 (34.2)
**Septic shock on admission, n (%)**	54 (13.6)	28 (27.2)	32 (17.1)
BMI *, kg/m^2^	26.1 [24.0–29.4]	25.7 [23.1–29.3]	25.5 [23.1–29.3]
**BMI classification, n (%)**			
Underweight	9 (2.3)	5 (4.9)	9 (4.8)
Normal	141 (35.5)	38 (36.9)	79 (42.2)
Overweight	162 (40.8)	40 (38.8)	65 (34.8)
Obese	85 (21.4)	20 (19.4)	34 (18.2)
**BMI < 25 kg/m^2^, n (%)**	164 (41.3)	46 (44.7)	90 (48.1)
ICU LOS *, days	11 [6–19]	14 [8–23]	12 [6–20]
Hospital LOS *, days	19 [12–31]	23 [15–33]	22 [13–32]
**ICU mortality, n (%)**	124 (31.2)	65 (63.1)	78 (41.7)
**28 days mortality, n (%)**	96 (24.2)	51 (49.5)	62 (33.2)
**Hospital mortality, n (%)**	133 (33.5)	68 (66.0)	84 (44.9)
mNUTRIC score *	3 [2–5]		
NRS 2002 *	3 [3–4]		
MUST score *	4 [4–4]		

* median, [IQR]. mNUTRIC: Modified Nutrition Risk in the Critically Ill score, NRS 2002: Nutritional Risk Screening 2002, MUST: Malnutrition Universal Screening Tool, ECOG: Eastern Cooperative Oncology Group, CFS: clinical frailty scale, APACHE: acute physiology and chronic health evaluation, SOFA: sequential organ failure assessment, PaO_2_/FiO_2_: Partial pressure of oxygen/Fraction of inspired oxygen, IMV: invasive mechanical ventilation, AKI: acute kidney injury, BMI: body mass index, ICU: intensive care unit, LOS: length of stay.

**Table 2:** Association between Nutrition Assessment Tools and ICU Mortality.

	Sensitivity	Specificity	*p* Value
**mNUTRIC ≥ 1**	1.00	0.06	<0.01
**mNUTRIC ≥ 2**	0.99	0.26	
**mNUTRIC ≥ 3**	0.90	0.49	
**mNUTRIC ≥ 4**	**0.77**	**0.74**	
**mNUTRIC ≥ 5**	0.52	0.86	
**mNUTRIC ≥ 6**	0.35	0.60	
**mNUTRIC ≥ 7**	0.19	0.99	
**mNUTRIC ≥ 8**	0.08	0.99	
**mNUTRIC ≥ 9**	0.04	1.00	
**mNUTRIC = 10**	0.01	1.00	
**NRS 2002 ≥ 2**	1.00	0.01	<0.01
**NRS 2002 ≥ 3**	0.88	0.21	
**NRS 2002 ≥ 4**	**0.63**	**0.60**	
**NRS 2002 ≥ 5**	0.17	0.45	
**NRS 2002 = 6**	0.07	0.98	
**MUST ≥ 2**	1.00	0.00	0.04
**MUST ≥ 3**	**0.21**	**0.91**	
**MUST ≥ 4**	0.11	0.95	
**MUST ≥ 5**	0.08	0.95	
**MUST = 6**	0.04	0.99	

ICU: intensive care unit, mNUTRIC: Modified Nutrition Risk in the Critically Ill score, NRS 2002: Nutritional Risk Screening 2002, MUST: Malnutrition Universal Screening Tool.

**Table 3:** Characteristics and outcomes of patients according to nutritional risk scores’ thresholds.

	mNUTRIC < 4 (n = 231)	mNUTRIC ≥ 4 (n = 166)	*p* Value	NRS 2002 < 4 (n = 210)	NRS 2002 ≥ 4 (n = 187)	*p* Value	MUST < 3 (n = 347)	MUST ≥ 3 (n = 50)	*p* Value
Age *, years	59 [51–67]	75 [66–82]	**<0.01**	57 [51–63]	77 [71–82]	**<0.01**	65 [55–76]	66 [53–74]	0.77
**Male sex, n (%)**	157 (68.0)	97 (58.4)	0.06	137 (65.2)	117 (62.6)	0.32	226 (65.1)	28 (56.0)	0.21
ECOG *	1 [0–2]	2 [1–3]	**<0.01**	1 [0–2]	2 [1–3]	**<0.01**	1 [0–2]	2 [1–3]	**<0.01**
CFS *	2 [1–4]	6 [4–7]	**<0.01**	2 [1–4]	5 [3–7]	**<0.01**	3 [2–6]	6 [3–7]	**<0.01**
APACHE II score *	12 [10–14]	20 [17–24]	**<0.01**	13 [10–16]	17 [14–21]	**<0.01**	14 [11–18]	17 [14–21]	**<0.01**
Admission SOFA score *	3 [2–4]	5 [4–8]	**<0.01**	3 [2–4]	4 [3–6]	**<0.01**	4 [2–5]	4 [3–7]	**0.02**
PaO_2_/FiO_2_ on admission *	164 [120–240]	138 [108–194]	**<0.01**	148 [111–225]	154 [118–230]	0.66	149 [112–225]	156 [125–232]	0.56
BMI *, kg/m^2^	26.1 [24.2–29.4]	26.0 [23.4–29.4]	0.21	26.9 [24.6–29.5]	25.5 [23.1–29.3]	**<0.01**	26.5 [24.3–29.5]	21.2 [19.0–25.2]	**<0.01**
**BMI classification, n (%)**									
Underweight	3 (1.3)	6 (3.6)	0.49	0 (0)	9 (4.8)	**<0.01**	0 (0)	9 (18.0)	**<0.01**
Normal	82 (35.5)	59 (35.5)	0.42	62 (29.5)	79 (42.2)	**<0.01**	113 (32.6)	28 (56.0)	**<0.01**
Overweight	95 (41.1)	67 (40.4)	0.33	97 (46.2)	65 (34.8)	**<0.01**	153 (44.1)	9 (18.0)	**<0.01**
Obese	51 (22.1)	34 (20.5)	0.35	51 (24.3)	34 (18.2)	**<0.01**	81 (23.3)	4 (8.0)	**0.01**
**BMI <25 kg/m^2^, n (%)**	86 (37.2)	68 (41.0)	0.25	64 (30.5)	90 (48.1)	**<0.01**	117 (33.7)	37 (74.0)	**<0.01**
**AKI on admission, n (%)**	36 (15.6)	76 (45.8)	**<0.01**	48 (22.9)	64 (34.2)	**<0.01**	96 (27.7)	16 (32.0)	0.52
**Septic shock on admission, n (%)**	19 (8.2)	35 (21.1)	**<0.01**	22 (10.5)	32 (17.1)	**0.03**	38 (11.0)	16 (32.0)	**<0.01**
**IMV on admission, n (%)**	54 (23.4)	108 (65.1)	**<0.01**	72 (34.3)	90 (48.1)	**<0.01**	134 (38.6)	28 (56.0)	**0.02**
Duration of IMV, days *	12 [5–22]	12 [5–21]	0.68	10 [4–21]	13 [7–22]	**<0.01**	12 [6–21]	11 [2–26]	0.41
**ICU mortality, n (%)**	29 (12.6)	95 (57.2)	**<0.01**	46 (21.9)	78 (41.7)	**<0.01**	97 (28.0)	27 (54.0)	**<0.01**
**28 days mortality, n (%)**	21 (9.1)	75 (45.2)	**<0.01**	34 (16.2)	62 (33.2)	**<0.01**	76 (21.9)	20 (40.0)	**<0.01**
**Hospital mortality, n (%)**	34 (14.7)	99 (59.6)	**<0.01**	49 (23.3)	84 (44.9)	**<0.01**	103 (29.7)	30 (60.0)	**<0.01**
ICU LOS *, days	9 [5–15]	14 [7–23]	**<0.01**	10 [6–18]	12 [6–20]	0.06	11 [6–19]	10 [5–19]	0.71
Hospital LOS *, days	17 [11–29]	23 [15–35]	**<0.01**	17 [11–29]	22 [13–32]	**<0.01**	19 [12–31]	21 [12–39]	0.28

* median, [IQR]. Text in bold with *p* values indicates statistical significance. mNUTRIC: Modified Nutrition Risk in the Critically Ill score, NRS 2002: Nutritional Risk Screening 2002, MUST: Malnutrition Universal Screening Tool, ECOG: Eastern Cooperative Oncology Group, CFS: clinical frailty scale, APACHE: acute physiology and chronic health evaluation, SOFA: sequential organ failure assessment, PaO_2_/FiO_2_: Partial pressure of oxygen/Fraction of inspired oxygen, IMV: invasive mechanical ventilation, AKI: acute kidney injury, BMI: body mass index, ICU: intensive care unit, LOS: length of stay.

**Table 4:** Laboratory values * of patients on ICU admission according to nutritional risk scores’ thresholds.

	mNUTRIC < 4 (n = 231)	mNUTRIC ≥ 4 (n = 166)	*p* Value	NRS 2002 < 4 (n = 210)	NRS 2002 ≥ 4 (n = 187)	*p* Value	MUST < 3 (n = 347)	MUST ≥ 3 (n = 50)	*p* Value
**Haemoglobin,** g/dL	13.1 [11.1–14.1]	11.5 [9.8–13.4]	**<0.01**	13.0 [10.7–14.1]	12.1 [10.1–13.7]	**<0.01**	12.9 [10.8–17.0]	12.7 [10.5–14.0]	**<0.01**
**Platelet,** (×10^3^)	190 [149–276]	185 (129–256)	0.05	191 [146–272]	187 [137–265]	0.48	192 [144–270]	189 [142–270]	0.06
**Lymphocyte** (×10^3^)	0.7 [0.5–1.1]	0.6 [0.4–1.0]	0.08	0.7 [0.5–1.1]	0.6 [0.4–1.0]	0.11	0.7 [0.5–1.0]	0.7 [0.5–1.1]	0.55
**NLR**	8.5 [4.0–14.3]	10.1 [4.4–20.0]	0.07	8.1 [4.0–14.7]	10.1 [4.5–19.5]	0.06	8.8 [4.1–17.0]	8.8 [4.1–17.0]	0.97
**Prealbumin,** mg/dL	13 [9–18] (n = 157)	11 [8–15] (n = 135)	**<0.01**	13 [10–18] (n = 141)	11 [8–15] (n = 151)	**<0.01**	12 [9–17] (n = 124)	11 [9–17] (n = 168)	0.08
**Albumin,** g/dL	3.3 [3.0–3.6]	3.0 [2.7–3.4]	**<0.01**	3.3 [3.0–3.6]	3.1 [2.7–3.5]	**<0.01**	3.3 [2.9–3.6]	3.2 [2.8–3.5]	**0.01**
**Total protein,** g/dL	6.3 [5.8–6.8]	6.1 [5.5–6.6]	**<0.01**	6.2 [5.7–6.7]	6.2 [5.6–6.7]	0.30	6.3 [5.8–6.8]	6.2 [5.7–6.7]	**0.04**
**BUN,** mg/dL	17.7 [13.8–23.1]	28.7 [19.2–48.3]	**<0.01**	17.5 [13.5–24.7]	24.6 [18.0–40.4]	**<0.01**	21.0 [14.6–31.6]	21.0 [14.7–31.8]	0.50
**Creatinine,** mg/dL	0.8 [0.6–1.0]	1.1 [0.8–1.6]	0.25	0.8 [0.7–1.0]	1.0 [0.7–1.3]	**<0.01**	0.9 [0.7–1.2]	0.9 [0.7–1.2]	0.76
**CRP,** mg/L	7.7 [2.0–14.1]	8.5 [3.9–15.8]	0.05	7.8 [2.1–14.4]	8.3 [3.3–15.0]	0.51	8.2 [2.7–15.0]	8.0 [2.7–14.8]	0.65
**IL-6,** pg/mL	34 [12–90] (n = 65)	56 [29–125] (n = 44)	**0.02**	35 [15–114] (n = 56)	55 [24–96] (n = 53)	0.68	40 [16–100] (n = 63)	42 [16–113] (n = 46)	0.53
**Procalcitonin,** ng/mL	0.21 [0.10–0.45]	0.47 [0.14–1.91]	**<0.01**	0.25 [0.10–0.67]	0.32 [0.11–1.22]	**<0.01**	0.22 [0.09–0.72]	0.25 [0.10–0.80]	**0.02**

* median, [IQR]. Text in bold with *p* values indicates statistical significance. NLR: neutrophil to lymphocyte ratio, CRP: C-Reactive protein, BUN: blood urea nitrogen. IL-6: interleukin-6, BUN: blood urea nitrogen.

**Table 5:** Multivariate analysis for predicting ICU mortality.

Parameters	Odds Ratio (95% Confidence Interval)	*p*
**A**		
Malignancy	3.53 (1.45–8.58)	**<0.01**
CFS	1.23 (1.03–1.45)	**0.02**
IMV on admission	44.12 (17.99–108.17)	**<0.01**
mNUTRIC ≥ 4	1.49 (1.23–1.88)	**0.02**
**B**		
Malignancy	3.81 (1.55–9.34)	**<0.01**
CFS	1.24 (1.05–1.47)	**<0.01**
IMV on admission	43.21 (17.90–104.34)	**<0.01**
NRS 2002 ≥ 4	1.03 (0.42–2.54)	0.94
**C**		
Malignancy	3.43 (1.39–8.47)	**<0.01**
CFS	1.23 (1.04–1.45)	**0.02**
IMV on admission	43.31 (17.97–104.39)	**<0.01**
MUST ≥ 3	1.90 (0.67–5.33)	0.22

Text in bold with *p* values indicated statistical significance. ICU: intensive care unit, CFS: Clinical Frailty Scale, IMV: invasive mechanical ventilation, mNUTRIC: Modified Nutrition Risk in the Critically Ill score, NRS 2002: Nutritional Risk Screening 2002, MUST: Malnutrition Universal Screening Tool. **MODEL A**: Adjusted for age, presence of hypertension, presence of cardiac disease, acute physiology and chronic health evaluation (APACHE) II score, partial pressure of oxygen/Fraction of inspired oxygen ratio (PaO_2_/FiO_2_ ratio), presence of septic shock and presence of acute kidney injury. Nagelkerke R^2^: 0.68, Hosmer and Lemeshow Test: 0.63. **MODEL B**: Adjusted for age, acute physiology and chronic health evaluation (APACHE) II score, partial pressure of oxygen/Fraction of inspired oxygen ratio (PaO_2_/FiO_2_ ratio), presence of septic shock and presence of acute kidney injury. Nagelkerke R^2^: 0.68, Hosmer and Lemeshow Test: 0.41. **MODEL C**: Adjusted for age, acute physiology and chronic health evaluation (APACHE) II score, partial pressure of oxygen/Fraction of inspired oxygen ratio (PaO_2_/FiO_2_ ratio), presence of septic shock and presence of acute kidney injury. Nagelkerke R^2^: 0.68, Hosmer and Lemeshow Test: 0.57.

## Data Availability

The original contributions presented in the study are included in the article, further inquiries can be directed to the corresponding authors.

## Appendix A

In this Appendix, we showed the characteristics and outcomes (Table A1) and laboratory values on ICU admission (Table A2) of ICU survivors and non-survivors.

**Table A1:** Characteristics and outcomes of ICU survivors and non-survivors.

	Total (n = 397)	Survivors (n = 273)	Non-Survivors (n = 124)	*p* Value
**Age *, years**	65 [55–76]	62 [54–73]	70 [60–82]	**<0.01**
**Male sex, n (%)**	254 (64.0)	171 (62.6)	83 (66.9)	0.24
**Comorbidities, n (%)**				
Hypertension	209 (52.6)	134 (49.1)	75 (60.1)	**0.02**
Diabetes	136 (34.3)	93 (34.1)	43 (34.7)	0.50
Cardiac disease	134 (33.8)	81 (29.7)	53 (42.7)	**<0.01**
Malignancy	77 (19.4)	33 (12.1)	44 (35.5)	**<0.01**
Chronic lung disease	66 (16.6)	45 (16.5)	21 (16.9)	0.51
Chronic kidney disease	35 (8.8)	21 (7.7)	14(11.3)	0.16
**ECOG ***	1 [0–2]	1 [0–2]	2 [1–3]	**<0.01**
**CFS ***	3 [2–6]	3 [1–4]	6 [4–7]	**<0.01**
**APACHE II score ***	15 [11–19]	13 [10–17]	20 [16–25]	**<0.01**
**Admission SOFA score ***	4 [2–5]	3 [2–4]	6 [4–8]	**<0.01**
**PaO_2_/FiO_2_ on admission ***	150 [113–226]	165 [120–244]	134 [105–183]	**<0.01**
**IMV on admission, n (%)**	162 (40.8)	48 (17.6)	114 (91.9)	**<0.01**
**Duration of IMV, days ***	12 [5–22]	9 [5–22]	13 [5–22]	0.92
**AKI on admission, n (%)**	112 (28.2)	51 (18.7)	61 (49.2)	**<0.01**
**Septic shock on admission, n (%)**	54 (13.6)	18 (6.6)	36 (29.0)	**<0.01**
**BMI *, kg/m^2^**	26.1 [24.0–29.4]	26.5 [24.2–29.4]	25.7 [23.4–29.3]	0.09
**BMI classification, n (%)**				
Underweight	9 (2.3)	4 (1.5)	5 (4.0)	0.11
Normal	141 (35.5)	91 (33.3)	50 (40.3)	0.10
Overweight	162 (40.8)	114 (41.8)	48 (38.8)	0.32
Obese	85 (21.4)	64 (23.4)	21 (16.9)	0.09
**BMI < 25 kg/m^2^, n (%)**	164 (41.3)	97 (35.5)	67 (46.0)	**0.03**
**mNUTRIC score ***	3 [2–5]	3 [1–4]	5 [4–6]	**<0.01**
**NRS 2002 ***	3 [3–4]	3 [3–4]	4 [3–4]	**<0.01**
**MUST score ***	2 [2–2]	2 [2–2]	2 [2–2]	**<0.01**
**ICU LOS *, days**	11 [6–19]	9 [5–15]	15 [8–25]	**<0.01**
**Hospital LOS *, days**	19 [12–31]	13 [7–17]	19 [12–28]	**<0.01**
**ICU mortality, n (%)**	124 (31.2)			
**28 days mortality, n (%)**	96 (24.2)			
**Hospital mortality, n (%)**	133 (33.5)			

* median, [IQR]. Text in bold with *p* values indicates statistical significance. ECOG: Eastern Cooperative Oncology Group, CFS: clinical frailty scale, APACHE: acute physiology and chronic health evaluation, SOFA: sequential organ failure assessment, PaO_2_/FiO_2_: Partial pressure of oxygen/Fraction of inspired oxygen, IMV: invasive mechanical ventilation, AKI: acute kidney injury, ICU: intensive care unit, LOS: length of stay, NLR: neutrophyl to lymphocyte ratio.

**Table A2:** Laboratory values * on ICU admission.

	Total (n = 397)	Survivors (n = 273)	Non-Survivors (n = 124)	*p* Value
**Haemoglobin,** g/dL	12.7 [10.5–14.0] (n = 397)	12.9 [10.8–14.1] (n = 273)	11.5 [9.7–13.6] (n = 124)	**<0.01**
**Platelet,** (×10^3^)	189 [142–270] (n = 397)	197 [154–275] (n = 273)	171 [114–252] (n = 124)	**0.03**
**Lymphocyte** (×10^3^)	0.69 [0.45–1.06] (n = 397)	0.73 [0.50–1.07] (n = 273)	0.59 [0.35–0.98] (n = 124)	**0.01**
**NLR**	8.8 [4.2–17.0] (n = 397)	8.3 [4.0–13.7] (n = 273)	12.3 [4.4–23.4] (n = 124)	**0.04**
**Prealbumin,** mg/dL	11.6 [8.6–16.9] (n = 292)	12.0 [9.0–18.0] (n = 190)	11.0 [8.0–16.0] (n = 102)	**<0.01**
**Albumin,** g/dL	3.20 [2.80–3.54] (n = 397)	3.36 [3.00–3.84] (n = 273)	3.01 [2.59–3.44] (n = 124)	**<0.01**
**Total protein,** g/dL	6.20 [5.69–6.74] (n = 397)	6.3 [5.8–9.8] (n = 273)	6.0 [5.5–6.6] (n = 124)	**0.04**
**Triglycerides,** mg/dL	134 [101–193] (n = 146)	126 [100–193] (n = 114)	146 [111–199] (n = 32)	0.37
**BUN,** mg/dL	21.0 [14.7–31.8] (n = 397)	18.7 [14.0–25.3] (n = 273)	27.6 [18.0–53.7] (n = 124)	**<0.01**
**Creatinine,** mg/dL	0.86 [0.66–1.22] (n = 397)	0.80 [0.64–1.06] (n = 273)	1.03 [0.77–1.55] (n = 124)	**<0.01**
**CRP,** mg/L	8.0 [2.7–14.8] (n = 397)	8.3 [2.2–14.9] (n = 273)	7.8 [3.9–14.3] (n = 124)	0.62
**IL-6,** pg/mL	42.0 [15.9–112.5] (n = 109)	36 [13–94] (n = 79)	65 [31–121] (n = 30)	**0.01**
**Ferritin,** mL/ng	441 [194–915] (n = 397)	421 [189–809] (n = 273)	597 [213–1300] (n = 124)	**<0.01**
**Procalcitonin,** ng/mL	0.24 [0.09–0.81] (n = 397)	0.19 [0.08–0.57] (n = 273)	0.33 [0.14–1.79] (n = 124)	**<0.01**

* median, [IQR]. Text in bold with *p* values indicates statistical significance. NLR: neutrophil to lymphocyte ratio, CRP: C-Reactive protein, IL-6: interleukin-6, BUN: blood urea nitrogen.

## References

[1] Correia M.I.T.D., Waitzberg D.L. (2003). The Impact of Malnutrition on Morbidity, Mortality, Length of Hospital Stay and Costs Evaluated through a Multivariate Model Analysis. Clin. Nutr..

[2] Su Lim L., Ong K.C.B., Chan Y.H., Loke W.C., Ferguson M., Daniels L. (2012). Malnutrition and Its Impact on Cost of Hospitalization, Length of Stay, Readmission and 3-Year Mortality. Clin. Nutr..

[3] Lew C.C.H., Yandell R., Fraser R.J.L., Chua A.P., Chong M.F.F., Miller M. (2017). Association Between Malnutrition and Clinical Outcomes in the Intensive Care Unit: A Systematic Review. J. Parenter. Enter. Nutr..

[4] Cummings M.J., Baldwin M.R., Abrams D., Jacobson S.D., Meyer B.J., Balough E.M., Aaron J.G., Claassen J., Rabbani L.E., Hastie J. (2020). Epidemiology, Clinical Course, and Outcomes of Critically Ill Adults with COVID-19 in New York City: A Prospective Cohort Study. Lancet.

[5] Halaçli B., Kaya A., Topeli A. (2020). Critically Ill COVID-19 Patient. Turk. J. Med. Sci..

[6] Grasselli G., Zangrillo A., Zanella A., Antonelli M., Cabrini L., Castelli A., Cereda D., Coluccello A., Foti G., Fumagalli R. (2020). Baseline Characteristics and Outcomes of 1591 Patients Infected With SARS-CoV-2 Admitted to ICUs of the Lombardy Region, Italy. JAMA.

[7] Docherty A.B., Harrison E.M., Green C.A., Hardwick H.E., Pius R., Norman L., Holden K.A., Read J.M., Dondelinger F., Carson G. (2020). Features of 20 133 UK Patients in Hospital with Covid-19 Using the ISARIC WHO Clinical Characterisation Protocol: Prospective Observational Cohort Study. BMJ.

[8] Yang X., Yu Y., Xu J., Shu H., Xia J., Liu H., Wu Y., Zhang L., Yu Z., Fang M. (2020). Clinical Course and Outcomes of Critically Ill Patients with SARS-CoV-2 Pneumonia in Wuhan, China: A Single-Centered, Retrospective, Observational Study. Lancet. Respir. Med..

[9] Rahman S., Montero M.T.V., Rowe K., Kirton R., Kunik F. (2021). Epidemiology, Pathogenesis, Clinical Presentations, Diagnosis and Treatment of COVID-19: A Review of Current Evidence. Expert. Rev. Clin. Pharmacol..

[10] Bakouny Z., Hawley J.E., Choueiri T.K., Peters S., Rini B.I., Warner J.L., Painter C.A. (2020). COVID-19 and Cancer: Current Challenges and Perspectives. Cancer. Cell.

[11] Guan W., Liang W., Zhao Y., Liang H., Chen Z., Li Y., Liu X., Chen R., Tang C., Wang T. (2020). Comorbidity and Its Impact on 1590 Patients with COVID-19 in China: A Nationwide Analysis. Eur. Respir. J..

[12] Rahman S., Singh K., Dhingra S., Charan J., Sharma P., Islam S., Jahan D., Iskandar K., Samad N., Haque M. (2020). The Double Burden of the COVID-19 Pandemic and Polypharmacy on Geriatric Population—Public Health Implications. Ther. Clin. Risk. Manag..

[13] Ortiz-Prado E., Simbaña-Rivera K., Gómez- Barreno L., Rubio-Neira M., Guaman L.P., Kyriakidis N.C., Muslin C., Jaramillo A.M.G., Barba-Ostria C., Cevallos-Robalino D. (2020). Clinical, Molecular, and Epidemiological Characterization of the SARS-CoV-2 Virus and the Coronavirus Disease 2019 (COVID-19), a Comprehensive Literature Review. Diagn. Microbiol. Infect. Dis..

[14] Short K.R., Kedzierska K., van de Sandt C.E. (2018). Back to the Future: Lessons Learned From the 1918 Influenza Pandemic. Front. Cell. Infect. Microbiol..

[15] Czapla M., Juárez-Vela R., Gea-Caballero V., Zieliński S., Zielińska M. (2021). The Association between Nutritional Status and In-Hospital Mortality of COVID-19 in Critically-Ill Patients in the ICU. Nutrients.

[16] Martinuzzi A.L.N., Manzanares W., Quesada E., Reberendo M.J., Baccaro F., Aversa I., Kecskes C.E., Magnífico L., González V., Bolzico D. (2021). Nutritional Risk and Clinical Outcomes in Critically Ill Adult Patients with COVID-19. Nutr. Hosp..

[17] Alikiaii B., Heidari Z., Fazeli A., Rahimi Varposhti M., Moradi Farsani D., Fattahpour S., Rafiee S., Bagherniya M. (2021). Evaluation of the Effectiveness of the Nutritional Risk Screening System 2002 (NRS-2002) in COVID-19 Patients Admitted to the Intensive Care Unit. Int. J. Clin. Pract..

[18] Jia H. (2016). Pulmonary Angiotensin-Converting Enzyme 2 (ACE2) and Inflammatory Lung Disease. Shock.

[19] Huang C., Wang Y., Li X., Ren L., Zhao J., Hu Y., Zhang L., Fan G., Xu J., Gu X. (2020). Clinical Features of Patients Infected with 2019 Novel Coronavirus in Wuhan, China. Lancet.

[20] Guan W.-J., Ni Z.-Y., Hu Y., Liang W.-H., Ou C.-Q., He J.-X., Liu L., Shan H., Lei C.-L., Hui D.S.C. (2020). Clinical Characteristics of Coronavirus Disease 2019 in China. N. Engl. J. Med..

[21] Rahman A., Hasan R.M., Agarwala R., Martin C., Day A.G., Heyland D.K. (2016). Identifying Critically-Ill Patients Who Will Benefit Most from Nutritional Therapy: Further Validation of the “Modified NUTRIC” Nutritional Risk Assessment Tool. Clin. Nutr..

[22] Kondrup J., Rasmussen H.H., Hamberg O., Stanga Z., Ad Hoc ESPEN Working Group (2003). Nutritional Risk Screening (NRS 2002): A New Method Based on an Analysis of Controlled Clinical Trials. Clin. Nutr..

[23] Stratton R.J., Hackston A., Longmore D., Dixon R., Price S., Stroud M., King C., Elia M. (2004). Malnutrition in Hospital Outpatients and Inpatients: Prevalence, Concurrent Validity and Ease of Use of the “Malnutrition Universal Screening Tool” (‘MUST’) for Adults. Br. J. Nutr..

[24] Heyland D.K., Dhaliwal R., Jiang X., Day A.G. (2011). Identifying Critically Ill Patients Who Benefit the Most from Nutrition Therapy: The Development and Initial Validation of a Novel Risk Assessment Tool. Crit. Care.

[25] Mukhopadhyay A., Henry J., Ong V., Leong C.S.-F., Teh A.L., van Dam R.M., Kowitlawakul Y. (2017). Association of Modified NUTRIC Score with 28-Day Mortality in Critically Ill Patients. Clin. Nutr..

[26] Cederholm T., Jensen G.L., Correia M.I.T.D., Gonzalez M.C., Fukushima R., Higashiguchi T., Baptista G., Barazzoni R., Blaauw R., Coats A. (2019). GLIM Criteria for the Diagnosis of Malnutrition—A Consensus Report from the Global Clinical Nutrition Community. Clin. Nutr..

[27] Maciel L.R.M.d.A., Franzosi O.S., Nunes D.S.L., Loss S.H., Dos Reis A.M., Rubin B.d.A., Vieira S.R.R. (2019). Nutritional Risk Screening 2002 Cut-Off to Identify High-Risk Is a Good Predictor of ICU Mortality in Critically Ill Patients. Nutr. Clin. Pract..

[28] Vong T., Yanek L.R., Wang L., Yu H., Fan C., Zhou E., Oh S.J., Szvarca D., Kim A., Potter J.J. (2022). Malnutrition Increases Hospital Length of Stay and Mortality among Adult Inpatients with COVID-19. Nutrients.

[29] Liu G., Zhang S., Mao Z., Wang W., Hu H. (2020). Clinical Significance of Nutritional Risk Screening for Older Adult Patients with COVID-19. Eur. J. Clin. Nutr..

[30] de Vries M.C., Koekkoek W.K., Opdam M.H., van Blokland D., van Zanten A.R. (2018). Nutritional Assessment of Critically Ill Patients: Validation of the Modified NUTRIC Score. Eur. J. Clin. Nutr..

[31] Singer P., Blaser A.R., Berger M.M., Alhazzani W., Calder P.C., Casaer M.P., Hiesmayr M., Mayer K., Montejo J.C., Pichard C. (2019). ESPEN Guideline on Clinical Nutrition in the Intensive Care Unit. Clin. Nutr..

[32] Barazzoni R., Bischoff S.C., Breda J., Wickramasinghe K., Krznaric Z., Nitzan D., Pirlich M., Singer P., Endorsed by the ESPEN Council (2020). ESPEN Expert Statements and Practical Guidance for Nutritional Management of Individuals with SARS-CoV-2 Infection. Clin. Nutr..

[33] Singer M., Deutschman C.S., Seymour C.W., Shankar-Hari M., Annane D., Bauer M., Bellomo R., Bernard G.R., Chiche J.-D., Coopersmith C.M. (2016). The Third International Consensus Definitions for Sepsis and Septic Shock (Sepsis-3). JAMA.

[34] (2012). Clinical Practice Guidelines for Acute Kidney Injury. https://kdigo.org/guidelines/acute-kidney-injury/.

[35] Body Mass Index—BMI. https://www.who.int/data/gho/data/themes/topics/topic-details/GHO/body-mass-index.

[36] Youden W.J. (1950). Index for Rating Diagnostic Tests. Cancer.

[37] Jensen G.L., Compher C., Sullivan D.H., Mullin G.E. (2013). Recognizing Malnutrition in Adults: Definitions and Characteristics, Screening, Assessment, and Team Approach. JPEN J. Parenter. Enteral. Nutr..

[38] Zhang H., Shao B., Dang Q., Chen Z., Zhou Q., Luo H., Yuan W., Sun Z. (2021). Pathogenesis and Mechanism of Gastrointestinal Infection With COVID-19. Front. Immunol..

[39] Wełna M., Adamik B., Kübler A., Goździk W. (2023). The NUTRIC Score as a Tool to Predict Mortality and Increased Resource Utilization in Intensive Care Patients with Sepsis. Nutrients.

[40] McClave S.A., Martindale R.G., Vanek V.W., McCarthy M., Roberts P., Taylor B., Ochoa J.B., Napolitano L., Cresci G., A.S.P.E.N. Board of Directors (2009). Guidelines for the Provision and Assessment of Nutrition Support Therapy in the Adult Critically Ill Patient: Society of Critical Care Medicine (SCCM) and American Society for Parenteral and Enteral Nutrition (A.S.P.E.N.). JPEN J. Parenter. Enteral. Nutr..

[41] Mendes R., Policarpo S., Fortuna P., Alves M., Virella D., Heyland D.K., Portuguese NUTRIC Study Group (2017). Nutritional Risk Assessment and Cultural Validation of the Modified NUTRIC Score in Critically Ill Patients-A Multicenter Prospective Cohort Study. J. Crit. Care.

[42] Wang N., Wang M.-P., Jiang L., Du B., Zhu B., Xi X.-M. (2021). Association between the Modified Nutrition Risk in Critically Ill (mNUTRIC) Score and Clinical Outcomes in the Intensive Care Unit: A Secondary Analysis of a Large Prospective Observational Study. BMC Anesthesiol..

[43] Jeong D.H., Hong S.-B., Lim C.-M., Koh Y., Seo J., Kim Y., Min J.-Y., Huh J.W. (2018). Comparison of Accuracy of NUTRIC and Modified NUTRIC Scores in Predicting 28-Day Mortality in Patients with Sepsis: A Single Center Retrospective Study. Nutrients.

[44] Tseng C.-C., Tu C.-Y., Chen C.-H., Wang Y.-T., Chen W.-C., Fu P.-K., Chen C.-M., Lai C.-C., Kuo L.-K., Ku S.-C. (2021). Significance of the Modified NUTRIC Score for Predicting Clinical Outcomes in Patients with Severe Community-Acquired Pneumonia. Nutrients.

[45] Chen M., Wang S.-A., Yang J., Bai J., Gu J., Luo H., Zhang X., Han Y., Shao J., Xu Y. (2024). Association of Systemic Immune-Inflammation Index with Malnutrition among Chinese Hospitalized Patients: A Nationwide, Multicenter, Cross-Sectional Study. Front. Nutr..

[46] Cortés-Aguilar R., Malih N., Abbate M., Fresneda S., Yañez A., Bennasar-Veny M. (2024). Validity of Nutrition Screening Tools for Risk of Malnutrition among Hospitalized Adult Patients: A Systematic Review and Meta-Analysis. Clin. Nutr..

[47] Prakash J., Verma S., Shrivastava P., Saran K., Kumari A., Raj K., Kumar A., Ray H.N., Bhattacharya P.K. (2024). Modified NUTRIC Score as a Predictor of All-Cause Mortality in Critically Ill Patients: A Systematic Review and Meta-Analysis. Indian J. Crit. Care Med..

[48] Zhou F., Yu T., Du R., Fan G., Liu Y., Liu Z., Xiang J., Wang Y., Song B., Gu X. (2020). Clinical Course and Risk Factors for Mortality of Adult Inpatients with COVID-19 in Wuhan, China: A Retrospective Cohort Study. Lancet.

[49] Kalaiselvan M.S., Renuka M.K., Arunkumar A.S. (2017). Use of Nutrition Risk in Critically Ill (NUTRIC) Score to Assess Nutritional Risk in Mechanically Ventilated Patients: A Prospective Observational Study. Indian J. Crit. Care Med..

[50] Cattani A., Eckert I.C., Brito J.E., Tartari R.F., Silva F.M. (2020). Nutritional Risk in Critically Ill Patients: How It Is Assessed, Its Prevalence and Prognostic Value: A Systematic Review. Nutr. Rev..

